# Fostering In-Service Teachers’ Motivation, Professional Competence, and Implementation of Physically Active Learning via Example-Based, Experience-Based, or Problem-Solving Professional Development Formats

**DOI:** 10.3390/ejihpe16030042

**Published:** 2026-03-12

**Authors:** Tjari Klimpki, Tim Heemsoth

**Affiliations:** 1 Movement, Games and Sports Education, Faculty of Education, Universität Hamburg, 20146 Hamburg, Germany; 2 Sports Pedagogy, Institute of Sports Science, Europa-Universität Flensburg, 24943 Flensburg, Germany

**Keywords:** physically active learning, professional development, experience-based learning, example-based learning, motivation, professional competence, implementation, randomized experimental design

## Abstract

Physically Active Learning (PAL) integrates physical activity into classroom teaching and has been shown to benefit students’ cognitive, social, and academic outcomes. Despite these advantages, PAL is not yet sustainably implemented in everyday school practice, highlighting the need for effective professional development (PD) formats for teachers. This randomized controlled experimental study examined how different PD formats, varying in their mode of engagement with ready-to-use PAL materials, affect teachers’ motivation, professional competence, and implementation of PAL. A total of 153 in-service primary teachers participated in a 2.5 h PD training and were randomly assigned to one of three formats: example-based learning, experience-based learning, or problem-solving. Data were collected at pre-test, post-test, and a six-week follow-up using standardized questionnaires. Results showed that teachers in the experience-based format reported significantly higher motivation during the PD training than those in the other formats. Across all formats, attitude and self-efficacy regarding PAL increased over time, whereas no significant gains in knowledge were observed. No significant differences between PD formats regarding overall implementation of PAL were observed. Exploratory analyses indicated a potential advantage of the experience-based format. Overall, the findings suggest that immersive, experience-based PD formats may be particularly effective in fostering teachers’ motivation.

## 1. Introduction

Physically Active Learning (PAL) refers to the integration of physical activity into classroom teaching across all curriculum subjects (Daly-Smith et al., 2020; Teslo et al., 2023). It encompasses two main forms: *learning with movement* and *learning through movement* (Hildebrandt-Stramann et al., 2017; Russ et al., 2017; Teslo et al., 2023). Learning with movement includes (i) movement breaks, (ii) physically active teaching methods, and (iii) alternative learning arrangements or locations (Hildebrandt-Stramann et al., 2017; Russ et al., 2017). In these cases, movement serves a supportive yet content-independent function, such as an energizer, transition, or motivational element (Vazou et al., 2020). In contrast, (iv) learning through movement refers to instructional activities in which physical activity is directly linked to the learning content, allowing students to engage with and better understand the subject matter through embodied experience (Hildebrandt-Stramann et al., 2017; Laging, 2017; Russ et al., 2017). This fourfold distinction reflects a synthesis of international discourses on movement integration and PAL (Daly-Smith et al., 2020; Russ et al., 2017; Vazou et al., 2020) and provides a coherent framework for conceptualizing how physical activity can be pedagogically embedded in everyday classroom practice.

PAL is considered a highly beneficial concept for classroom teaching (Watson et al., 2017). A large body of research has demonstrated PAL’s positive effects on students’ concentration (Ma et al., 2015), social competencies (Riley et al., 2015), classroom atmosphere (Carson et al., 2014), and academic achievement (Donnelly & Lambourne, 2011). Moreover, PAL aligns with international educational initiatives such as the *Whole Child Approach* (ASCD, 2012) and the *Healthy School Framework* (WHO, 2020). Despite these advantages, on average, students continue to spend most of their school day being sedentary (Egan et al., 2019), with only limited opportunities for movement. This discrepancy suggests that PAL has not yet been sustainably implemented in everyday school practice.

The successful implementation of PAL largely depends on teachers, who play a central role in planning, structuring, and delivering instruction (Daly-Smith et al., 2020; Lerum et al., 2019). Therefore, teachers need to feel confident in their ability to apply PAL effectively in their teaching (Daly-Smith et al., 2021; Teslo et al., 2023). Furthermore, they should be familiar with and know the diverse instructional formats and strategies that PAL entails. However, many teachers feel insufficiently competent or experienced to integrate PAL into their classroom practice (Daly-Smith et al., 2021; Teslo et al., 2023). This emphasizes the need for targeted professional development (PD) to enhance teachers’ confidence, attitude, and knowledge about PAL. Yet empirical research on effective PD formats, particularly those that promote innovative instructional approaches such as PAL, remains limited (Darling-Hammond et al., 2017). In addition to teachers’ attitude, self-efficacy, and knowledge, their motivation represents a key factor influencing both learning processes (Boekaerts, 2016) during PD and the subsequent implementation of innovations such as PAL (Schrader et al., 2020). Accordingly, the present study also examines teachers’ experienced motivation during the PD format as an outcome of different formats.

Concerning PAL, qualitative teacher interviews indicate that hands-on, ready-to-use examples, particularly comprising specific tasks in which pupils learn through movement, could be a helpful tool (Daly-Smith et al., 2021; McMullen et al., 2016). Based on these empirical findings, it appears reasonable to design PD formats that explicitly incorporate such materials. However, the form of engagement with such examples can vary considerably. To date, it remains unclear which *mode of engagement* and, consequently, which PD format best supports teachers’ motivation, attitude, self-efficacy, knowledge, and ultimately, implementation behavior. When designing such PD formats, at least two promising approaches with ready-to-use examples can be considered: (a) engagement through analyzing text-based teaching examples from an observational perspective (*example-based*; Heemsoth & Kleickmann, 2018), and (b) engagement through experiencing PAL lesson examples from a learner’s perspective (*experience-based*; Dewey, 1938; Girvan et al., 2016).

Against this background, the present study aims to systematically compare these two forms of engagement with (c) a third, *problem-solving*-based format that reflects more traditional PD formats (Opfer & Pedder, 2011). The study addresses the following research questions:

1. To what extent do different PD formats, which vary in terms of the mode of engagement with ready-to-use PAL materials, influence primary teachers’ motivation during the PD training?
2. To what extent do different PD formats affect primary teachers’ attitude, self-efficacy, and knowledge toward PAL?
3. To what extent do different PD formats affect primary school teachers’ implementation of PAL?

The following sections outline the theoretical and conceptual background of this study. Section 1.1 introduces the relevant competence facets required for PAL implementation. Section 1.2 elaborates on how different PD formats may foster these facets through varying modes of engagement with ready-to-use PAL materials. Finally, Section 1.3 presents the design and hypotheses of the current study.

### 1.1. Relevant Competence Facets for PAL

From a competence-based perspective on teacher professionalization (Baumert & Kunter, 2006; Blömeke et al., 2015; Kunter et al., 2011; Shavelson, 2013; Shulman, 2005), effective teaching requires the acquisition and continual development of professional competence, which in turn influences instructional performance and classroom practice (Kunter et al., 2011). According to the model of determinants of professional competence proposed by Kunter et al. (2011), learning opportunities for teachers, in interaction with personal characteristics and contextual conditions, influence teachers’ professional competence, which in turn shapes instructional behavior. This competence–performance linkage is theoretically grounded in the model and has received converging empirical support across instructional contexts (Blömeke et al., 2015; Weyers et al., 2023). Teacher professionalization is therefore understood as a dynamic, lifelong process shaped by formative experiences, reflective opportunities, and structured learning contexts (Blömeke et al., 2015). Within this framework (Kunter et al., 2011), learning opportunities for teachers can be conceptualized as structured PD trainings that aim to influence teachers’ professional competence and their instructional behavior through their impact on professional competence. Moreover, in-service PD training represents a particularly relevant phase for enhancing and expanding professional competence (OECD, 2009).

Teacher competence can be conceptualized through various theoretical models that differ in focus and scope (Baumert & Kunter, 2006; Blömeke et al., 2015; Kunter et al., 2011; Shavelson, 2013; Shulman, 2005). According to the well-established model by Baumert and Kunter (2006), professional teacher competence consists of four components: (a) motivational orientations, (b) self-regulatory skills, (c) value commitments and beliefs, and (d) professional knowledge. We assume that successful implementation of PAL in the classroom requires competence in all these areas.

Synthesizing perspectives from implementation and professionalization research suggests that distinct competence components shape teachers’ implementation of PAL. First, attitude toward PAL, understood as teachers’ beliefs in the effectiveness and potential benefits of PAL (Baumert & Kunter, 2006; Durlak & DuPre, 2008), plays a decisive role in whether teachers are willing to adopt and sustain PAL in their teaching. Second, self-efficacy for implementing PAL, rooted in Bandura’s (1997) social cognitive theory and understood as a part of self-regulatory skills (Baumert & Kunter, 2006), refers to teachers’ confidence in their ability to plan, conduct, and evaluate PAL lessons effectively (Doll & Meyer, 2021; Schrader et al., 2020). Third, knowledge about PAL is essential for understanding both the pedagogical rationale and the practical implementation of PAL activities (Durlak & DuPre, 2008; Schrader et al., 2020). Accordingly, teachers need to distinguish between learning with movement and learning through movement (see Section 1), and understand how each can contribute to student learning and engagement (Hildebrandt-Stramann et al., 2017; van Gog et al., 2019). In sum, the present study focuses on three central competence components relevant to teachers’ professionalization regarding PAL: (1) Attitude toward PAL, (2) Self-efficacy for implementing PAL, and (3) Knowledge about PAL.

### 1.2. Fostering Teachers’ Professional PAL Competence via Different Modes of Engagement with Ready-to-Use Materials

Teachers’ professional competence regarding PAL can be strengthened through structured opportunities encouraging to engage with instructional, ready-to-use materials in ways that are closely aligned with classroom practice (Daly-Smith et al., 2021; Stylianou et al., 2016). However, it remains unclear which mode of engagement with such positive examples of PAL most effectively fosters teachers’ attitude, self-efficacy, knowledge, and ultimately their classroom implementation of PAL.

Research on teacher PD has identified several core design principles associated with positive effects on teacher learning and instructional practice (Sims et al., 2025). Meta-analyses and large-scale reviews consistently highlight the importance of content focus, active learning opportunities, coherence with teachers’ instructional context, collective participation, and sufficient duration (Darling-Hammond et al., 2017; Sims et al., 2025). More recently, Sims et al. (2025) proposed and meta-analytically tested a theory-driven model of effective PD, arguing that successful programs must address four core purposes: providing insight into teaching and learning processes, building motivation to change, developing instructional techniques, and embedding new practices through structured rehearsal and contextualized application (IMTP framework). Across 104 randomized controlled trials, PD programs integrating mechanisms across all four functions yielded larger student-level effects than those addressing only isolated components. However, while this body of research provides valuable insights into structural features and mechanisms of effective PD, comparatively fewer investigations have systematically examined how different instructional engagement formats within PD differentially influence specific competence components.

Regarding PAL specifically, existing research has primarily focused on feasibility, implementation barriers, and teachers’ perceptions of single-format interventions (Daly-Smith et al., 2021; Pulling Kuhn et al., 2021). Although these studies provide important descriptive, qualitative, and practice-oriented insights, experimental or quasi-experimental comparisons of distinct modes of engagement with PAL materials remain scarce. Consequently, it is still insufficiently understood whether and how different ways of working with ready-to-use PAL examples may differentially foster teachers’ motivation, professional competence, and subsequent implementation behavior.

In this study, we focus on two primary modes of engagement. First, teachers can engage with PAL materials in a text-based, example-oriented manner (Renkl, 2014; van Gog et al., 2019), reflecting on detailed lesson examples without direct physical participation. This example-based learning approach is well-established in educational research, demonstrating that structured observation and analysis of modeled solutions support professional knowledge acquisition and can enhance the attitude of pupils and pre-service teachers (Heemsoth, 2019; Heemsoth & Kleickmann, 2018; Renkl, 2014; van Gog et al., 2019). In the context of in-service PAL implementation, text-based engagement may allow teachers to study ready-to-use PAL tasks in detail, understand their pedagogical rationale, and mentally rehearse implementation strategies. Example-based learning has the advantage of efficiency and accessibility (van Gog & Rummel, 2010), particularly for short-term PD formats.

Second, teachers can experience PAL lessons from a learner’s perspective, participating actively in ready-to-use instructional settings themselves. This experience-based approach (Dewey, 1938) offers high immersion, combining direct embodied engagement with reflection on pedagogical intentions. From a learning-theoretical perspective, this aligns with experiential learning frameworks, which emphasize the role of active, contextually grounded experiences in promoting deep understanding and long-term retention (Dewey, 1938; Girvan et al., 2016; Ray et al., 2013). This approach appears especially promising for PAL, as learning through movement itself can be understood as a form of experience-based learning (Dewey, 1938; Hildebrandt-Stramann et al., 2017). By experiencing PAL from the pupils’ point of view, we assume that teachers can internalize both the content and the procedural aspects of PAL, enhancing their confidence to implement similar activities independently.

Both example-based and experience-based approaches can be contrasted with problem-solving learning, a more traditional instructional format (Opfer & Pedder, 2011) in which teachers are introduced to new concepts and, compared to example-based or experience-based approaches, then apply the new knowledge to novel scenarios more directly (Newell & Simon, 1972; van Gog et al., 2019). Within problem-solving, teachers generate solutions themselves rather than observing or participating in modeled examples. According to example-based research, as problem-solving provides less direct exposure to concrete PAL practices, it may be less effective for initial competence acquisition, particularly in short-term PD formats. This potential disadvantage is often attributed in cognitive load theory to excessive cognitive load during unguided problem-solving (Heemsoth & Kleickmann, 2018; Sweller & Cooper, 1985). While this mechanism is well-established in experimental learning research (Renkl, 2014; Seidel et al., 2013), its applicability to short-term in-service PD formats for PAL has not yet been directly tested. In sum, these three modes of engagement represent distinct strategies that differ theoretically in their cognitive and motivational affordances for developing teachers’ professional competence in PAL. However, empirical comparisons of their relative effectiveness in in-service PAL contexts remain scarce. Comparing these approaches allows the study to address the currently unresolved question of how different modes of engagement with ready-to-use PAL materials influence teachers’ motivation, attitude, self-efficacy, knowledge, and classroom implementation.

### 1.3. The Current Study

From a learning-theoretical perspective, motivation toward the learning content constitutes a central precondition for professional learning (Boekaerts, 2016) and competence development. Within the competence model of Baumert and Kunter (2006), motivational orientations are conceptualized as an integral component of professional competence, shaping teachers’ engagement and persistence in learning activities. Implementation research similarly underscores that motivation is crucial not only for the sustained transfer of innovations into practice but also for the learning processes that precede it (Durlak & DuPre, 2008; Schrader et al., 2020). In the context of PAL-focused PD, motivation determines the depth of teachers’ cognitive and affective engagement with the new instructional approach. As Boekaerts (2016) argues, motivation regulates learners’ goal-directed behavior, therefore assumed the implementation behavior. Thus, teachers’ situational motivation during a PD training can theoretically be conceptualized as a pivotal affective variable that facilitates or constrains the acquisition of PAL-related competencies and their later classroom implementation. Empirical research in both student learning (Kriegbaum et al., 2018) and teacher professional development (Schrader et al., 2020) suggests that higher motivation is associated with deeper engagement and sustained transfer, although direct causal pathways in short-term PD contexts remain underexplored.

Taken together, the present study is guided by a sequential conceptual model linking professional development formats to classroom implementation via motivational and competence-related processes. Specifically, different modes of engagement with ready-to-use PAL materials are assumed to shape teachers’ situational motivation during the PD training, which in turn may influence the depth of cognitive engagement and subsequent competence development. Building on competence models of teacher professionalization (Kunter et al., 2011), changes in attitude, self-efficacy, and knowledge are expected to function as proximal determinants of instructional performance, here operationalized as PAL implementation. Moreover, given the short duration of the intervention, potential effects are expected to be modest and possibly more pronounced for motivational and belief-related components than for professional knowledge. The study, therefore, tests format-specific differences across these interconnected, yet theoretically distinct, outcome domains.

Beyond this background, this study is based on three research questions.

1. To what extent do different PD formats, which vary in terms of the mode of engagement with ready-to-use PAL materials, influence primary teachers’ motivation during the PD training?

We expect that teachers in the experience-based learning format will report the highest levels of motivation. This expectation is grounded in the immersive nature of the format (Dewey, 1938), which allows teachers to engage with PAL from a learner’s perspective and interact with peers in an active setting. Moreover, research on PAL has shown that learning through movement is associated with higher learner motivation (Ma et al., 2015). As the experience-based PD format itself incorporates learning through movement, it can be assumed that teachers may experience similar motivational benefits. In addition, PAL itself has been described by students as “something fun” (Quarmby et al., 2024), suggesting that teachers may perceive it similarly.

2. To what extent do different PD formats affect primary teachers’ attitude, self-efficacy, and knowledge toward PAL?

We assume that all three PD formats (example-based learning, experience-based, and problem-solving) will lead to measurable gains in attitude, self-efficacy, and knowledge toward PAL. Drawing on previous findings from example-based learning research (Renkl, 2014; van Gog et al., 2019) and empirical studies in pre-service teacher education (Heemsoth, 2019; Heemsoth & Kleickmann, 2018), we expect teachers in the example-based learning condition to show significantly greater gains compared to the problem-solving condition. Given that teachers have reported a preference for ready-to-use materials (Daly-Smith et al., 2021; McMullen et al., 2016), it is further hypothesized that the experience-based format will also outperform the problem-solving format in terms of competence development.

3. To what extent do different PD formats affect primary school teachers’ implementation of PAL?

We expect teachers in the example-based and experience-based learning formats to implement PAL more frequently than those in the problem-solving format. Empirical evidence suggests that the use of examples, either text-based or directly experienced, tends to be more effective than problem-solving alone (Heemsoth & Kleickmann, 2018; Renkl, 2014; van Gog et al., 2019). As knowledge is a prerequisite for implementation (Kunter et al., 2011), these advantages may plausibly translate into greater classroom implementation. However, the strength and immediacy of this transfer in short-duration PD interventions remain empirical questions.

From a design-based perspective, we expect differences in implementation, especially for the facet learning through movement, as the mode of engagement with ready-to-use materials varied exclusively for this facet across PD formats. However, potential format differences in the implementation of the other PAL facets cannot be ruled out. Increases in teachers’ motivation, attitude, and self-efficacy toward PAL, which may vary between formats, may theoretically function as mediating mechanisms that indirectly foster the implementation of PAL more broadly. These mediational pathways, however, are not directly tested in the present study and should therefore be interpreted cautiously.

## 2. Methods

### 2.1. Design

We conducted this ethically approved study as a randomized controlled experimental trial with a three-format design (example-based, experience-based, and problem-solving) and followed a pre–post-follow-up structure. Participants were randomly assigned to the three conditions at the individual level. The intervention consisted of a 2.5 h PD training on implementing PAL in the classroom teaching of primary schools. We collected data using online questionnaires at pre-test (T1) measuring socio-demographic variables, attitude, self-efficacy, and knowledge before the PD training, at post-test (T2) measuring motivation, attitude, self-efficacy, and knowledge immediately after the PD training and at follow-up-test (T3) six weeks after the training measuring implementation frequency, attitude, self-efficacy, and knowledge. While at T1 and T2 the survey was completed on site, for T3 the teachers received an email invitation and completed the survey online from home.

### 2.2. Participants

The final sample comprised 100 in-service certified primary teachers who participated in the intervention and completed all three measurement points (pre-test, post-test, and follow-up). Initially, 153 teachers took part in at least one measurement occasion. Accordingly, analyses relying on data collected up to post-test (T2), such as the motivation analysis, are based on the larger T2 sample (*N* = 153), whereas longitudinal analyses across all three measurement points rely on the final complete-case sample (*N* = 100). For the knowledge outcome, two additional cases had to be excluded due to missing data, resulting in a sample size of *N* = 98 for this specific analysis.

Participants were randomly assigned on an individual level to one of the three PD formats; however, format membership was assessed at post-test (T2). Consequently, teachers who participated only in pre-test (T1) could not be assigned to an experimental format and are therefore not reflected in the format-specific sample sizes at different measurement time points (see Table A1).

Attrition differed across the experimental conditions, already with higher sample mortality in the example-based format compared to the other formats between T1 and T2. Between T2 and T3, attrition rates amounted to 46% in the example-based format, 31% in the experience-based format, and 30% in the problem-solving format (see Table A1). As a result, the final sample consisted of 22 teachers in the example-based format, 38 teachers in the experience-based format, and 40 teachers in the problem-solving format.

To ensure baseline equivalence, χ^2^ tests of independence and one-way ANOVAs were conducted for all pre-test variables. No statistically significant differences between the format-groups were found regarding gender, age, teaching experience, attitude (T1), self-efficacy (T1), or knowledge regarding PAL (T1) (all *p*s > 0.05), indicating that the format-groups did not differ systematically before the intervention (see Table A2).

To examine whether attrition was systematically related to participant characteristics or study variables, additional dropout analyses were conducted. Teachers who completed all three measurement points were compared to those who dropped out with respect to gender, age, teaching experience, and all pre-test variables (motivation, attitude, self-efficacy, and knowledge at T1). No statistically significant differences were found between completers and dropouts (all *p*s > 0.05; see Table A3), suggesting that attrition was not systematically associated with observed baseline characteristics. In addition, a χ^2^-test indicated that dropout was not significantly associated with PD format (*p* = 0.237), although descriptively higher dropout rates were observed in the example-based format.

### 2.3. Procedure

We conducted a total of nine PD trainings between May 2024 and January 2025 at German primary schools. The PD training began with a pre-test (20 min). This was followed by an input session (25 min) covering various rationales for implementing PAL and an overview of its four facets (see Section 1). Subsequently, participants were assigned based on seating order: they were numbered (1–2–3) and grouped accordingly into one of the three experimental conditions (experience-based, example-based, or problem-solving), in which they engaged with ready-to-use PAL materials focusing on learning through movement in depth for 60 min. Following this phase, the post-test was conducted (20 min). As a conclusion, the central insights were summarized. To enhance intervention fidelity, all PD sessions were conducted by the same trained facilitators and followed a standardized script outlining the structure, content, and timing of each format. However, no formal fidelity checks or observational adherence measures were implemented. Teachers were encouraged to intensify their practical application of PAL in the following six weeks. Six weeks after the PD training, participants received an email intervention and conducted the follow-up test at home.

### 2.4. Experimental Intervention

**Figure 1:**
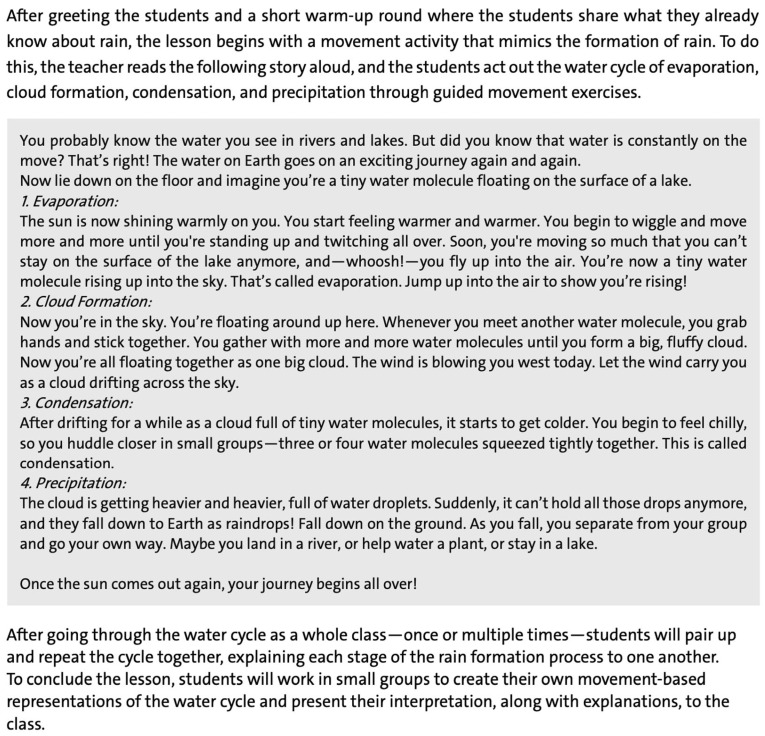
Science and social studies example as text-based material for the example-based format.

In all three formats, teachers worked on the same four instructional cases from the subjects of Mathematics, German, Science and Social Studies, and English. The mode the teachers engaged with the instructional cases differed depending on the format: In the example-based format, we provided participants with written, fully developed lesson plans that incorporated PAL, particularly learning through movement, for each of the four instructional cases (see Figure 1 showing the instructional case in Science and Social Studies). For example, their task for the science and social studies example was:

“The following describes a science and social studies lesson for a 4th-grade class, in which students explore the question: How does rain form? (1) Highlight the sections in the text where physically active learning takes place. (2) Explain how physically active learning is specifically implemented in the science and social studies lesson described below. In your explanation, distinguish between learning with movement and learning through movement.”

In the experience-based format, under the guidance of an instructor, participants actively experienced the four instructional cases from the students’ perspective by participating directly in the PAL scenarios. For the Science and Social Studies example, the procedure was as follows: First, the instructor provided teachers with contextual information regarding the subject, grade level, and topic. Second, they received the following instruction: “I will now read you a movement story. Take on the role of a water molecule. Whenever the water molecules perform a movement in the story, you should imitate it.” Third, they were instructed: “In pairs, go through the cycle two more times. At each station, explain the processes of rain formation to one another.” Fourth, they were asked: “In small groups, design your own cycle with original movement representations. Prepare it in such a way that you can present it to the class at the end of the lesson, including your explanations.”

In the problem-solving format, we asked participants to independently design a rough lesson plan for the given scenarios, aiming to incorporate PAL as extensively as possible, particularly learning through movement. This format thus engaged in self-directed development of PAL arrangements. For example, their task for the Science and Social Studies example was as follows:

“Develop a rough lesson plan that implements physically active learning, particularly learning through movement, for the following context. Subject: Science and Social Studies. Grade level: 4. Topic: How does rain form?”

Participants in all formats worked on each instructional case for 15 min, resulting in a total duration of 60 min.

### 2.5. Measures

We assessed motivation after the workshop phase using an eight-item scale from Rakoczy et al. (2005), which includes items such as “During the teacher training I attended last hour, time flew by”. The scale demonstrated very good reliability (α = 0.87).

We assessed teachers’ attitude toward PAL by combining items of two scales assessing the perceived benefits of PAL on the one hand and beliefs about PAL on the other hand. For the perceived benefits of PAL, we used six items that we adjusted according to Thiel et al. (2013). An example item is, “I am convinced that Physically Active Learning creates lasting experiences.” According to beliefs about PAL, we used seven items, partly self-developed and partly adapted and translated from Cook et al. (2018). One example item reads, “Physically Active Learning is a valuable didactic approach”. The resulting 13-item scale demonstrated very good reliability (T1: α = 0.86; T2: α = 0.87; T3: α = 0.88).

We measured self-efficacy for implementing PAL using an eight-item scale developed for this study, inspired by Doll and Meyer (2021). A typical item from this scale is “I feel confident in integrating Physically Active Learning into my teaching”. The scale demonstrated very good reliability (T1: α = 0.87; T2: α = 0.87; T3: α = 0.89). The scales assessing teachers’ motivation, attitude, and self-efficacy were answered on a four-point Likert scale ranging from “strongly disagree” (0) to “strongly agree” (3).

**Figure 2:**
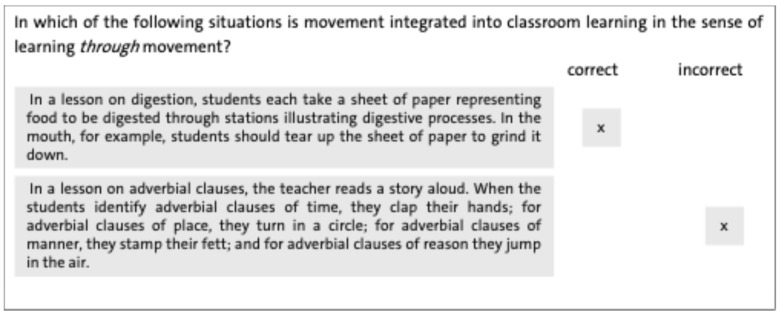
Two sample items used to operationalize knowledge about PAL.

The knowledge assessment focused on two key aspects: teachers’ understanding of the potential benefits of PAL, and their ability to distinguish between learning with movement and learning through movement (see Figure 2 for two sample items). The whole scale consists of 16 items. Participants were given different situations in classroom teaching. They were asked to identify in which situations movement was integrated as learning through movement within a school context. Each item was coded as either correct or incorrect, with correct responses scored as 1 and incorrect ones as 0. The scale demonstrated acceptable reliability (T1: α = 0.69; T2: α = 0.72; T3: α = 0.79).

To assess the frequency of PAL implementation during the six teaching weeks following the PD training by the teachers, at T3 we asked how often they had implemented (a) movement breaks, (b) physically active teaching methods, (c) alternative learning arrangements or locations, and (d) learning through movement during the last six teaching weeks. Responses were given on a four-point scale: (1) ‘not at all,’ (2) ‘once to three times,’ (3) ‘at least four times and up to twice per week,’ and (4) ‘at least in every second lesson.’

### 2.6. Data Analysis

We conducted the statistical analyses using IBM SPSS Statistics 29. To examine to what extent different PD formats (example-based, experience-based, problem-solving) affect the teachers’ motivation during the PD training, we used a one-way ANOVA, where the self-reported motivation at T2 served as the dependent variable. For all significant main effects, we applied Bonferroni-adjusted post hoc tests to identify specific format differences.

To examine to what extent the different PD formats lead to significant changes in teachers’ attitude, self-efficacy, and knowledge toward PAL, we used repeated measures ANOVAs with subsequent simple effects analyses to compare the formats over time. The between-subject factor was format, with three levels (example-based, experience-based, problem-solving), and the within-subject factor was time, with three levels (T1, T2, T3). To test for the effect of formats, we specifically looked at the interaction of format x time. A significant interaction would indicate different learning gains within the three formats. We used simple effects analyses to test for the effect of time on all formats separately. In addition to complete-case rmANOVAs, we conducted sensitivity analyses using linear mixed-effects models with maximum likelihood estimation to include all available observations. F-tests for fixed effects were based on Type III sums of squares. Degrees of freedom were estimated using the Satterthwaite approximation, as implemented in SPSS.

Although linear mixed-effects models are generally robust to missing data under the assumption of missing at random, complete-case rmANOVAs were retained as the primary hypothesis-testing approach. This decision was guided by the experimental design, which focused on comparing format-specific change patterns across three clearly defined measurement points. The rmANOVA framework allows for a direct and transparent test of format × time interactions aligned with the study’s research questions. Attrition analyses indicated no systematic differences between completers and dropouts with respect to observed baseline characteristics (see Table A3), supporting the assumption that the complete-case approach would not introduce substantial bias. Linear mixed-effects models using maximum likelihood estimation were therefore conducted as sensitivity analyses to examine the robustness of the findings when including all available observations.

To examine to what extent the different PD formats affect the teachers’ implementation of PAL, we conducted a one-way MANOVA. We chose this approach to account for the intercorrelations between the four facets of PAL implementation (movement breaks, physically active teaching methods, alternative learning arrangements or locations, and learning through movement), which served as the dependent variables, and to control the familywise error rate at the multivariate level. We tested the assumption of homogeneity of covariance matrices using Box’s *M* test. Following the multivariate analysis, univariate one-way ANOVAs were inspected as follow-up tests to explore the impact of PD formats on each facet of PAL. For all significant main effects, we applied Bonferroni-adjusted post hoc tests to identify specific format differences.

Given the presence of multiple outcome variables, particular attention was paid to the control of Type I error. The analytic strategy was structured according to the three research questions, each representing a theoretically distinct outcome domain. Motivation (RQ1), the three professional competence facets (attitude, self-efficacy, and professional knowledge; RQ2), and the implementation facets (RQ3) were treated as separate conceptual domains rather than as a single family of dependent variables. Within each domain, appropriate adjustments were applied where necessary. Importantly, alpha adjustments were conducted within outcome domains rather than across all dependent variables globally, as the domains were derived from distinct theoretical constructs and addressed separate research questions. Although attitude, self-efficacy, and professional knowledge are often discussed under the broader umbrella of professional competence, they represent theoretically distinct facets with different psychological functions and developmental trajectories (Baumert & Kunter, 2006; Durlak & DuPre, 2008). Consequently, separate rmANOVAs were conducted for each facet rather than a multivariate repeated-measures analysis. This approach reflects the conceptual differentiation of the constructs and allows for facet-specific interpretation aligned with the theoretical framework guiding the study (Baumert & Kunter, 2006; Kunter et al., 2011).

We interpreted effect sizes using η^2^. According to Cohen’s (1988) guidelines, values of 0.01 < η^2^ < 0.09 indicate small effects, 0.09 < η^2^ < 0.25 medium effects, and η^2^ > 0.25 large effects.

## 3. Results

### 3.1. Research Question 1: Motivation

We conducted a one-way ANOVA to compare participants’ motivation during the PD training across the three PD formats. As motivation was assessed directly after the PD training, this analysis was based on the T2-sample (overall: *N* = 153, example-based: *N* = 41; experience-based: *N* = 55, problem-solving: *N* = 57).

A significant main effect of format was found (*F*(2, 150) = 14.39, *p* < 0.001, η^2^ = 0.16), indicating large format differences in reported motivation. Descriptive statistics revealed the highest motivation scores in the experience-based format (*M* = 2.38, *SD* = 0.44), followed by the problem-solving format (*M* = 2.01, *SD* = 0.66), and the lowest scores in the example-based format (*M* = 1.81, *SD* = 0.44).

Post hoc Bonferroni comparisons showed that participants in the experience-based format reported significantly higher motivation than those in both the example-based (*p* < 0.001) and problem-solving formats (*p* = 0.002).

In sum, the experience-based format was associated with significantly higher situational motivation compared to the other two formats.

### 3.2. Research Question 2: Teachers’ Competence

**Figure 3:**
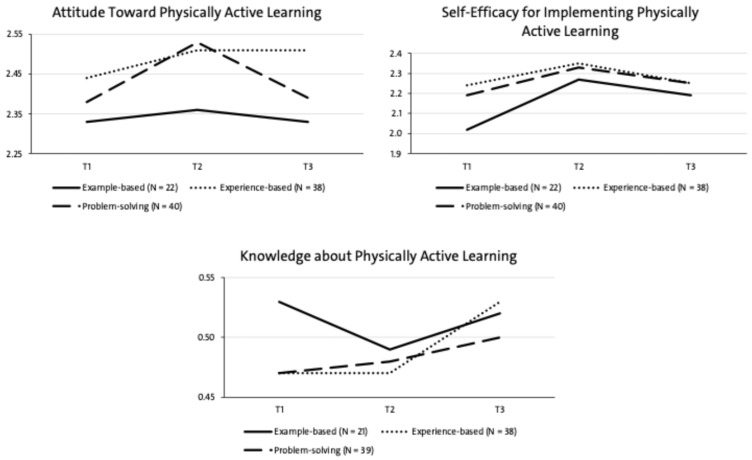
Development of Attitude, Self-Efficacy, and Knowledge Toward PAL among the Differentiated PD Formats over all Measurement Time Points.

We conducted rmANOVAs to examine the impact of the different formats on changes in teachers’ attitude (*N* = 100), self-efficacy (*N* = 100), and knowledge (*N* = 98) toward PAL. These analyses were based on the final sample of participants who completed all three measurements. Table 1 and Figure 3 present the means, standard deviations, and results of the rmANOVAs for all dependent variables across the three measurement time points and PD formats.

A significant main effect of time was found for attitude toward PAL (*F*(2, 198) = 4.44, *p* = 0.013, η^2^ = 0.044). Simple effects analyses showed that attitude improved significantly from pre- to post-test in the problem-solving format (*p* = 0.001). A significant main effect of time was also found for self-efficacy for implementing PAL (*F*(2, 198) = 5.91, *p* = 0.003, η^2^ = 0.057). Here, simple effect analyses showed that the example-based (*p* = 0.007) and the problem-solving format (*p* = 0.038) showed significant improvements from pre- to post-test. Yet, the main effect of time was not significant for knowledge about PAL (*F*(1.85, 179.06) = 2.46, *p* = 0.093, η^2^ = 0.025; Greenhouse-Geisser corrected, GG = 0.92).

Moreover, there were no significant facet × time interactions effect (attitude toward PAL: *F*(4, 194) = 1.27, *p* = 0.284, η^2^ = 0.025; self-efficacy for implementing PAL: *F*(4, 194) = 0.55, *p* = 0.696, η^2^ = 0.011; knowledge about PAL: *F*(3.69, 175.38) = 0.82, *p* = 0.507, η^2^ = 0.017; Greenhouse-Geisser corrected, *GG* = 0.92), indicating that changes over time did not differ significantly across the three PD formats.

Sensitivity analyses using linear mixed-effects models yielded substantively comparable results (see Table A4). The mixed-effects models included all available observations across measurement points (T1–T3). Across attitude, self-efficacy, and professional knowledge, the mixed models did not alter the pattern of statistical significance, nor did they reverse the direction of effects observed in the complete-case analyses. The overall conclusions regarding the absence of significant format × time interactions remained unchanged. These converging findings suggest that the results are robust across analytic approaches.

Overall, while attitude and self-efficacy increased over time, no competence facet showed differential development across PD formats, and knowledge did not change significantly over time.

### 3.3. Research Question 3: Implementation of PAL

To explore the extent to which teachers’ PAL implementation was affected by the different PD formats, we employed a one-way MANOVA. These analyses were based on the final sample of participants who completed all three measurements (overall: *N* = 102; example-based: *N* = 23; experience-based: *N* = 39; problem-solving: *N* = 40). Preliminary assumption testing revealed that the homogeneity of covariance matrices was met (Box’s *M* = 30.19, *p* = 0.103). The multivariate test served as the primary inferential basis in order to control Type I error across the correlated implementation outcomes.

The multivariate test yielded no significant effect of the PD format on the combined implementation (Wilks’ λ = 0.919, *F*(8, 192) = 1.04, *p* = 0.411, η^2^ = 0.041). Consequently, implementation behavior did not differ between formats.

Although the multivariate test was non-significant, follow-up ANOVAs were inspected for exploratory purposes. These indicated a potential effect only for the implementation of physically active teaching methods (*F*(2, 99) = 3.42, *p* = 0.037, η^2^ = 0.065). Post hoc Bonferroni comparisons suggest that participants in the experience-based format reported a higher implementation (*M* = 1.72, *SD* = 0.76) than those in the problem-solving format (*M* = 1.30, *SD* = 0.72; *p* = 0.031). No potential significant differences were observed between the experience-based and the example-based format (*M* = 1.52, *SD* = 0.59). However, in light of the non-significant multivariate result, this finding should be interpreted with caution.

For the implementation of all other PAL facets, no significant format differences were observed (implementation of movement breaks: *F*(2, 99) = 0.53, *p* = 0.593, η^2^ = 0.011; alternative learning arrangements or locations: *F*(2, 99) = 0.22, *p* = 0.803, η^2^ = 0.004; learning through movement: *F*(2, 99) = 1.15, *p* = 0.322, η^2^ = 0.023).

In summary, no multivariate format effect on implementation was detected, and only one exploratory univariate tendency emerged, which should be interpreted cautiously.

## 4. Discussion

This study examined the effects of three different PD formats (example-based, experience-based, and problem-solving) on teachers’ competence and implementation behavior regarding PAL. While the differences between the formats were generally small and often statistically non-significant, several noteworthy findings emerged that offer cautious and context-bound insights for the design of future PD formats. Importantly, for several key competence-related outcomes, no significant interaction effects between format and time were found, indicating that differential changes across formats were limited.

Regarding the first research question, the experience-based group reported significantly higher motivation during the PD training compared to the example-based and problem-solving formats. This finding is particularly relevant, as affective engagement is often considered a prerequisite for sustained learning and instructional change (Boekaerts, 2016). The immersive nature of the experience-based format may plausibly have contributed to conditions for deeper emotional involvement and future engagement with PAL. As these processes were not directly measured, this interpretation remains speculative. However, this effect was confined to motivation measured immediately after the PD session and does not imply broader differential development of professional competence across formats.

Concerning the second research question, all groups showed significant improvements in attitude toward PAL and self-efficacy for implementing PAL over time. These results underline that even short, focused PD trainings can positively influence self-regulatory skills as well as value commitments and beliefs components of professional competence (Baumert & Kunter, 2006; Durlak & DuPre, 2008; Schrader et al., 2020). Crucially, however, these improvements occurred across conditions and were not associated with significant format × time interactions, indicating that the data do not provide evidence for differential effectiveness of the formats with regard to these competence facets. However, no significant increase in professional knowledge about PAL was observed. This discrepancy may tentatively suggest that the PD formats primarily fostered teachers’ confidence and perceived understanding rather than actual knowledge gains. One plausible explanation could be that the training’s design emphasized the application and experience of PAL practices but did not provide sufficient depth or time for explicit knowledge construction. This mechanism was not directly assessed in the present study. This interpretation aligns with research suggesting that knowledge development requires iterative reflection and elaboration phases beyond initial exposure (Renkl, 2014; van Gog et al., 2019). In addition, the relatively short duration of the intervention may have limited opportunities for the consolidation and integration of conceptual knowledge. While motivational and belief-related components may respond more readily to brief interventions, the development of stable and transferable professional knowledge typically requires more sustained engagement and repeated cognitive elaboration (Kleickmann et al., 2013). Another possible, yet not directly tested, explanatory mechanism may be what has been described as an “illusion of understanding” (Kulgemeyer et al., 2022), that is, a situation in which exposure to well-structured examples increases perceived comprehension without corresponding gains in transferable knowledge. Nevertheless, given the absence of differential effects, this does not apply exclusively to the example-based format. Across all groups, the limited intervention time and focus on practical demonstration may have constrained opportunities for deeper cognitive processing. Furthermore, the absence of measurable knowledge gains may also be related to a potential misalignment between the instructional content emphasized during the PD and the operationalization of knowledge in the assessment instrument. Future research should therefore explicitly examine whether short-term PD formats differentially affect perceived versus actual knowledge gains. It may also be beneficial to explicitly align knowledge assessments with the specific learning objectives of the PD training to more comprehensively capture potential learning effects. Future PD formats could therefore benefit from incorporating more explicit scaffolding and reflection phases that translate experiential or modeled practice into conceptual understanding.

Regarding the third research question, the absence of a significant multivariate effect suggests that the mode of engagement with ready-to-use PAL materials did not substantially alter teachers’ overall implementation behavior across the four facets. Consequently, varying the instructional design of a short PD training alone may not be sufficient to shape broad differences in classroom implementation. This finding nuances the initial assumption that distinct forms of engagement would translate into differentiated behavioral outcomes. Although the multivariate effect was not significant, exploratory follow-up ANOVAs suggest a descriptive tendency for the implementation of physically active teaching methods, which should be interpreted with caution given the non-significant overall test. From a design-based perspective, this finding is noteworthy, as the PD formats differed exclusively in their mode of engagement with examples of *learning through movement*, whereas the input on physically active teaching methods was identical across groups. Thus, this observation may reflect influences common to all formats rather than format-specific effects, although this remains speculative.

Importantly, the comparatively weaker pattern observed for the problem-solving format should not be interpreted as evidence against the general effectiveness of problem-based approaches. Problem-solving and inquiry-oriented formats are known to impose relatively high cognitive demands and require sufficient time-on-task, prior knowledge, and iterative reflection phases to unfold their full instructional potential (Renkl, 2014; Sweller & Sweller, 2006). In the present study, however, the intervention was deliberately designed as a brief and practice-oriented PD session. Within such a highly time-restricted context, the open and cognitively demanding nature of the problem-solving format may have required more time or prior knowledge than was available, potentially limiting observable short-term effects. This interpretation is theoretically grounded but was not empirically tested within the present design.

Although exploratory analyses pointed a possible advantage of the experience-based format for physically active teaching methods, this isolated tendency should not be overgeneralized and does not constitute robust evidence of format superiority. Rather than format superiority, it may reflect that certain PAL facets, particularly those considered low-threshold and less complex forms of PAL (Hildebrandt-Stramann et al., 2017; Mavilidi et al., 2022), are more susceptible to such indirect motivational carryover effects. From a competence-performance perspective (Kunter et al., 2011), it is conceivable that short-term motivational differences, such as those observed for the experience-based format, could theoretically facilitate initial behavioral experimentation without necessarily resulting in systematic or comprehensive implementation change, although such mediational pathways were not directly examined in this study. Accordingly, the present findings point to the limits of brief PD interventions in generating differentiated behavioral outcomes and should be interpreted as tentative indications rather than conclusive evidence of format-specific effectiveness.

Nonetheless, all effects must be interpreted considering the short duration of the PD training (You et al., 2025). Accordingly, the present study can be understood as an exploratory first investigation under highly time-restricted PD conditions rather than as a definitive test of the comparative effectiveness of the three formats. Beyond, data collection relied on self-reports, which may be subject to social desirability and response biases (Arnold et al., 1985). This concern is particularly salient about the assessment of PAL implementation, which was measured exclusively through teachers’ retrospective self-reported frequency ratings six weeks after the intervention. Such measures may be influenced not only by social desirability (Arnold et al., 1985) but also by recall biases (Fan et al., 2025), potentially leading to over- or underestimation of actual classroom practices. Although efforts were made to standardize the intervention, the absence of formal fidelity checks represents a limitation. Future studies should incorporate structured adherence measures or observational fidelity protocols to more rigorously document implementation integrity. Finally, the follow-up period was relatively short, capturing only initial implementation behavior rather than long-term instructional change (Smit et al., 2019). Therefore, conclusions about sustained changes in teaching practice must be drawn with caution. In addition, differential attrition across conditions, particularly in the example-based format, should be considered when interpreting the findings. Although attrition analyses indicated no systematic differences between completers and dropouts with respect to observed baseline characteristics, the unequal dropout rates may nevertheless have resulted in a selective final sample. However, the consistency of findings across complete-case analyses and mixed-effects models further supports the robustness of the reported effects. Consequently, the internal validity of format comparisons cannot be assumed to be unaffected, and group differences should be interpreted cautiously.

Despite these limitations, the study offers several important implications and contributes novel insights to the literature on teacher PD for PAL. Notably, the inclusion of an experience-based PD format represents a significant innovation in the field. To our knowledge, this is the first empirical study to systematically examine the effects of an immersive, learner-perspective-based format in the context of in-service PAL training.

Given the structural constraints of the intervention, a replication study with a substantially extended PD duration (You et al., 2025), iterative learning cycles, and embedded reflection phases would be necessary to more rigorously test the comparative potential of the three engagement formats. The promising effect on motivation of the experience-based format indicates a potentially relevant avenue for further investigation, ideally employing longitudinal designs and observational measures of instructional practice to substantiate and extend the present findings. In particular, future research should incorporate observational methods, structured classroom observations (Kohake, 2024; Weston et al., 2023), or other objective indicators of implementation fidelity to more accurately capture how and to what extent PAL is enacted in everyday teaching practice.

Beyond its research implications, the present findings offer guidance for the design of scalable and practice-oriented PD programs for PAL. Rather than positioning example-based, experience-based, and problem-solving approaches as competing formats, the results suggest that they may serve complementary functions when strategically sequenced within a coherent PD structure.

For short-term formats (e.g., 2–3 h workshops), an experience-based entry phase may be a feasible option to foster initial engagement, based on the present findings. This can be followed by example-based modeling using ready-to-use PAL material packages, which provide structure, reduce preparation effort, and enhance teachers’ sense of feasibility (Daly-Smith et al., 2021; McMullen et al., 2016). Within such time-constrained formats, highly open problem-solving phases may require careful scaffolding, as their effectiveness likely depends on processing time and prior conceptual grounding (Renkl, 2014; Sweller & Sweller, 2006).

In full-day school-based PD settings (e.g., 6 h in-service training), a sequenced model appears especially promising: beginning with immersive experience-based elements to stimulate engagement, followed by structured example-based input to consolidate understanding, and culminating in guided problem-solving phases in which teachers collaboratively adapt PAL materials to their own classroom contexts. Such an approach allows both affective activation and deeper cognitive processing (van Gog et al., 2019) within a realistic institutional timeframe.

For more extensive, multi-module PD programs (e.g., certificate courses), the integration of iterative problem-solving cycles can become particularly relevant (Renkl, 2014; van Gog et al., 2019). Here, teachers could implement PAL materials between sessions, reflect on classroom experiences, and engage in structured peer-coaching dialogs. Embedding ready-to-use PAL material packages (Daly-Smith et al., 2021; McMullen et al., 2016) within such cycles may enhance scalability, while collegial exchange and feedback structures (Heise, 2009) can support sustained implementation and instructional refinement.

Taken together, the findings suggest that scalable PD programs may benefit less from choosing a single “most effective” format and more from intentionally combining complementary instructional approaches in alignment with available time resources and institutional conditions.

## Figures and Tables

**Table 1:** Means, Standard Deviations, and Results of Repeated Measures ANOVAs for Teachers’ Attitude, Self-Efficacy, and Knowledge Toward PAL Across Three Professional Development Formats and Measurement Time Points.

	Formats			rmANOVA				
	Example-based (*N* = 22) *M* (*SD*)	Experience-based (*N* = 38) *M* (*SD*)	Problem-solving (*N* = 40) *M* (*SD*)	*F*	*df1*	*df2*	*p*	η^2^
**Attitude toward Physically Active Learning**								
T1	2.33 (0.38)	2.44 (0.34)	2.38 (0.42)	1.27	4	194	0.284	0.025
T2	2.36 (0.39)	2.51 (0.31)	2.53 (0.38)					
T3	2.33 (0.33)	2.51 (0.36)	2.39 (0.39)					
**Self-Efficacy for implementing Physically Active Learning**								
T1	2.02 (0.45)	2.24 (0.51)	2.19 (0.49)	0.55	4	194	0.696	0.011
T2	2.27 (0.45)	2.35 (0.45)	2.33 (0.38)					
T3	2.19 (0.44)	2.25 (0.46)	2.25 (0.49)					
**Knowledge about Physically Active Learning**								
	Example-based (*N* = 21) *M* (*SD*)	Experience-based (*N* = 38) *M* (*SD*)	Problem-solving (*N* = 39) *M* (*SD*)					
T1	0.53 (0.14)	0.47 (0.13)	0.47 (0.16)	0.82	3.69 *	175.38 *	0.507	0.017
T2	0.49 (0.20)	0.47 (0.15)	0.48 (0.19)					
T3	0.52 (0.22)	0.53 (0.19)	0.50 (0.19)					

* Greenhouse-Geisser corrected; Mauchly’s Test of Sphericity *p* = 0.017, *GG* = 0.92.

## Data Availability

The data presented in this study are available from the corresponding author upon reasonable request. The data are not publicly available due to ethical and data protection considerations.

## Appendix A

**Table A1:** Sample Sizes by Experimental Group and Measurement Time Point (T1–T3).

Measurement Time Point	Overall	Example-Based	Experience-Based	Problem-Solving
T1	152	41	54	57
T2	153	41	55	57
T3	100	22	38	40

**Table A2:** Results of χ^2^-test of independence for Teachers’ Gender, and Results of ANOVAs for Teachers’ Teaching Experience, Age, Attitude, Self-Efficacy, and Knowledge Toward PAL Across Three PD Formats at Pre-Test (T1).

				**χ^2^-Test of Independence**				
	Example-based	Experience-based	Problem-solving	χ^2^		*df*	*p*	
**Gender**								
Divers (*N* = 1)	0	0	1	3.48		4	0.481	
Male (*N* = 11)	4	5	2					
Female (*N* = 141)	37	50	54					
				**ANOVA**				
Example-based *M* (*SD*)	Experience-based *M* (*SD*)		Problem-solving *M* (*SD*)	*F*	*df1*	*df2*	*p*	part. η^2^
**Teaching Experience**								
12.16 (8.70) (*N* = 38)	15.41 (9.78) (*N* = 45)		13.67 (10.47) (*N* = 55)	1.150	2	135	0.320	0.017
**Age**								
40.61 (10.67) (*N* = 41)	43.11 (12.13) (*N* = 55)		41.18 (10.66) (*N* = 57)	0.690	2	150	0.503	0.009
**Attitude Toward Physically Active Learning (T1)**								
2.29 (0.28) (*N* = 41)	2.42 (0.34) (*N* = 54)		2.36 (0.41) (*N* = 57)	1.470	2	149	0.233	0.019
**Self-Efficacy for Implementing Physically Active Learning (T1)**								
2.14 (0.53) (*N* = 41)	2.18 (0.49) (*N* = 54)		2.16 (0.50) (*N* = 57)	0.087	2	149	0.917	0.001
**Knowledge About Physically Active Learning (T1)**								
0.50 (0.15) (*N* = 41)	0.46 (0.15) (*N* = 52)		0.47 (0.16) (*N* = 55)	0.848	2	145	0.430	0.012

**Table A3:** Attrition analyses: Comparisons between dropouts and completers on demographic and pre-test variables, and association between dropout and PD format.

			**χ^2^-Test of Independence**		
	Dropout	non-Dropout	χ^2^	*df*	*p*
**Gender**					
Divers (*N* = 1)	1	0	2.050	2	0.359
Male (*N* = 11)	4	7			
Female (*N* = 140)	46	94			
**Format**					
Example-based (*N* = 41)	18	23	2.877	2	0.237
Experience-based (*N* = 54)	15	39			
Problem-solving (*N* = 57)	18	39			
			***t*** **-test**		
Dropout *M* (*SD*)	non-Dropout *M* (*SD*)		*t*	*df*	*p*
**Teaching Experience**					
16.25 (9.60) (*N* = 32)	17.10 (9.07) (*N* = 74)		0.436	104	0.664
**Age**					
40.69 (10.92) (*N* = 51)	42.07 (11.28) (*N* = 101)		0.722	150	0.472
**Motivation**					
1.96 (0.60) (*N* = 51)	2.15 (0.56) (*N* = 101)		1.910	150	0.058
**Attitude Toward Physically Active Learning (T1)**					
2.29 (0.37) (*N* = 51)	2.39 (0.38) (*N* = 100)		1.524	149	0.130
**Self-Efficacy for Implementing Physically Active Learning (T1)**					
2.12 (0.51) (*N* = 51)	2.18 (0.50) (*N* = 100)		0.779	149	0.437
**Knowledge About Physically Active Learning (T1)**					
0.47 (0.17) (*N* = 48)	0.48 (0.15) (*N* = 100)		0.528	146	0.599

**Table A4:** Results of Linear Mixed Models and Estimated Marginal Means for Attitude, Self-Efficacy, and Knowledge.

	Formats			F-Test				
	Example-based *M*	Experience-based *M*	Problem-solving *M*		*F*	*df1*	*df2*	*p*
**Attitude toward Physically Active Learning**								
T1	2.29	2.40	2.36	*Time*	7.264	2	258.32	<0.001
T2	2.32	2.50	2.48					
T3	2.32	2.49	2.36					
				*Time x Format*	1.523	4	257.96	0.196
**Self-Efficacy for implementing Physically Active Learning**								
T1	2.14	2.17	2.16	*Time*	13.017	2	258.58	<0.001
T2	2.32	2.36	2.31					
T3	2.24	2.26	2.23					
				*Time x Format*	0.091	4	258.08	0.985
**Knowledge about Physically Active Learning**								
T1	0.51	0.46	0.47	*Time*	2.932	2	261.88	0.055
T2	0.51	0.45	0.47					
T3	0.54	0.52	0.49					
				*Time x Format*	0.441	4	261.21	0.779
